# The Role of Human Milk Oligosaccharides and Probiotics on the Neonatal Microbiome and Risk of Necrotizing Enterocolitis: A Narrative Review

**DOI:** 10.3390/nu12103052

**Published:** 2020-10-06

**Authors:** Lila S. Nolan, Jamie M. Rimer, Misty Good

**Affiliations:** Department of Pediatrics, Division of Newborn Medicine, Washington University School of Medicine, St. Louis, MO 63110, USA; lilanolan@wustl.edu (L.S.N.); jireland@wustl.edu (J.M.R.)

**Keywords:** prematurity, newborn, necrotizing enterocolitis, microbiome, human milk oligosaccharide, probiotic, breast milk

## Abstract

Preterm infants are a vulnerable population at risk of intestinal dysbiosis. The newborn microbiome is dominated by *Bifidobacterium* species, though abnormal microbial colonization can occur by exogenous factors such as mode of delivery, formula feeding, and exposure to antibiotics. Therefore, preterm infants are predisposed to sepsis and necrotizing enterocolitis (NEC), a fatal gastrointestinal disorder, due to an impaired intestinal barrier, immature immunity, and a dysbiotic gut microbiome. Properties of human milk serve as protection in the prevention of NEC. Human milk oligosaccharides (HMOs) and the microbiome of breast milk are immunomodulatory components that provide intestinal homeostasis through regulation of the microbiome and protection of the intestinal barrier. Enteral probiotic supplements have been trialed to evaluate their impact on establishing intestinal homeostasis. Here, we review the protective role of HMOs, probiotics, and synbiotic combinations in protecting a vulnerable population from the pathogenic features associated with necrotizing enterocolitis.

## 1. Introduction

Necrotizing enterocolitis (NEC) affects the gastrointestinal tract of preterm infants and remains a leading cause of morbidity and mortality in neonatal intensive care units. NEC is characterized by an amplified and destructive inflammatory response to intestinal dysbiosis, resulting in tissue damage and a loss of intestinal barrier integrity. There is an inverse relationship between the risk of developing NEC and birthweight and gestational age, as less than 10% of infants that develop the disease are born at term gestation [1]. NEC is a devastating disease as it manifests in compromised gastrointestinal and neurodevelopmental outcomes in a vulnerable population as well as a high risk of mortality in infants requiring surgical intervention [2]. While the exact pathogenesis of NEC remains an intense area of investigation, it is known that breast fed infants are largely protected from developing NEC, strongly suggesting components present in breast milk are protective. Refining the composition of infant formula to include protective components is essential in the effort to prevent NEC in infants that are unable to receive breast milk.

Observations of infants with NEC have shown evidence of intestinal bacterial dysbiosis when compared to healthy infants, which has led to the hypothesis that inappropriate gut bacterial colonization serves as a contributing feature in the development of NEC [3,4,5]. Additionally, exposure to antibiotics or proton pump inhibitors have been linked to an increased risk of developing NEC, as both can modulate the microbiome [6,7]. It has long been known that the commensal composition of the gut of infants with NEC differs from that of healthy infants [8]. The advance of 16s rRNA sequencing to identify microbial species in the intestinal microbiome has supported the observation that preterm infants demonstrate a lower overall diversity of species of fecal bacteria. In particular, preterm infants can experience intestinal dysbiosis, which is a bloom of opportunistic and pathogenic bacteria present in their stool and can predispose them to infection and inflammation [9]. The decreased diversity of the intestinal microbiome is more pronounced in infants with NEC, notably, these infants have been shown to have an overabundance of Gammaproteobacterial species and a paucity of obligate anaerobic species [4,10].

The preterm infant gut microbial environment can also be disrupted by exogenous factors, including cesarean delivery, antibiotic use, and formula feeding [9]. Alterations in the preterm infant gut microbiota carries a theoretical risk of bacterial translocation and development of NEC and sepsis, whereas healthy microbiotal development reduces the risk of these complications [11,12]. Hence, a variety of exogenous and endogenous therapeutic strategies have been investigated to strengthen gut barrier function and prevent intestinal dysbiosis related to prematurity.

In this review, we discuss the benefits of prebiotics, specifically human milk oligosaccharides, and enteral probiotic supplements in premature infants and their protective role on the intestinal epithelial barrier, the gut microbiome, and prevention against morbidities and mortalities associated with preterm birth.

## 2. Human Milk Oligosaccharides and the Preterm Infant Microbiome

### 2.1. HMO Content in Breast Milk

Breast milk from mothers of preterm infants contains a multitude of immunomodulating components, including soluble immunoglobulin A (IgA), growth factors, and prebiotics. These factors have known protective mechanisms against intestinal dysbiosis, intestinal barrier dysfunction, and risk of NEC [13]. The benefits of breast milk on the intestine were demonstrated using an ex vivo culture of neonatal mouse and premature human derived enteroids, in which exposure to human breast milk increased growth and proliferation of the enteroids [14]. Human milk oligosaccharides (HMOs) are prebiotic substances present in high abundance in maternal breast milk. Notably, some commercial infant formulas have recently emerged to include supplementation with specific HMOs [15]. Over 100 different oligosaccharide structures have been identified and characterized in human milk, and compositional changes in their concentration vary with stage of lactation [16]. The HMO 2′-fucosyllactose (2′FL) has been identified as the most abundant HMO in breast milk [17]. Breast fed infants also have a higher urinary excretion of complex oligosaccharides, including lacto-*N*-tetraose (LNT), lacto-N-fucopentaose I and II, and difucosyl-LNT, whereas these are present in small concentrations in the urine of formula fed infants [18]. The presence of urinary oligosaccharides in breast fed infants suggests that the intestinal absorption from breast milk provides HMOs with an ability to generate both targeted and systemic effects.

The prebiotic character of HMOs and an exclusive human milk diet are helpful in modulating the infant gut microbiome [17]. *Bifidobacterium* species represent about 90% of the total infant microbiome in the first year [19], and the dominance of certain *Bifidobacteria* species in infancy has been associated with breast milk and certain human milk components, including HMOs [20,21]. Notably, HMOs experience selective consumption by *Bifidobacterium infantis* and other commensal organisms in the intestine, operating as carbon sources for these beneficial enteric organisms [22,23]. The growth of intestinal Bifidobacterial communities is also influenced by human milk glycans (HMGs), which are composed of HMOs and their related glycoconjugates [22]. Fucosyltransferase 2 (*FUT2*), also known as the secretor gene, is among a variety of genes that are involved in building HMGs. In a study of 44 mother and infant dyads, Lewis and colleagues determined the phenotypic and genotypic secretor status of mothers by quantitation of markers of 2′-fucosylated HMOs in expressed breast milk [22]. Secretor-fed infants had a greater abundance of *Bifidobacterial* and *Bacteroides* in an analysis of the gut microbiota (*p <* 0.05), further revealing the role of HMOs in the enrichment of the infant microbiome [22].

### 2.2. HMOs and the Preterm Intestinal Barrier

**Figure 1:**
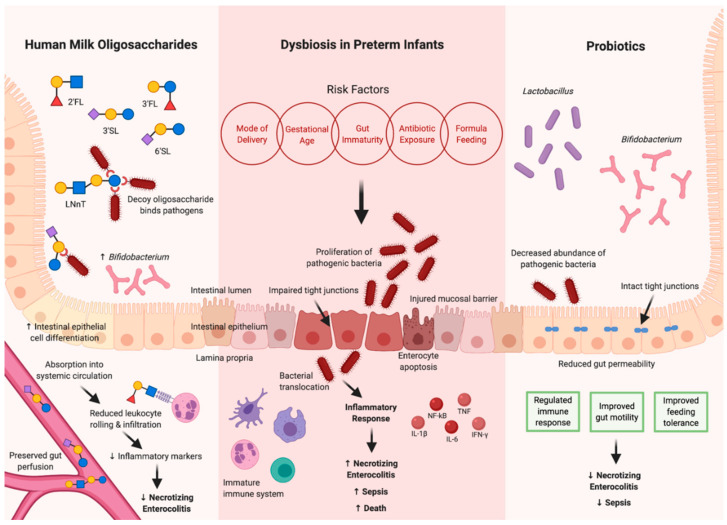
Effects of dysbiosis on the intestinal barrier in preterm infants and the protective effects of human milk oligosaccharides and enteral probiotics. Figure created with Biorender.com.

HMOs have a variety of established mechanisms for immunomodulatory protective effects on intestinal tissue (Figure 1). Intestinal barrier integrity is maintained by the epithelial cells and the tight junctions between the cells. In an in vitro epithelial model of the crypt–villus axis, treatment with a combination of HMO 3′sialyllactose (3′SL) and 6′sialyllactose (6′SL) resulted in decreased intestinal epithelial cell proliferation and increased differentiation, factors that can contribute to intestinal barrier maturation and integrity [24].

As intestinal barrier integrity can be compromised by enteric pathogens, targeted antimicrobial activity by HMOs has been an important field of investigation. HMOs have structural moieties that resemble the cell surface glycans targeted by enteric pathogens [25]. This similarity to host cell surface glycans effectively allows HMOs to operate as soluble decoy receptors that prevent the adhesion of enteric pathogens, including *Campylobacter jejuni* and Rotavirus, to target cells within the intestinal epithelium [25,26,27]. Additionally, human milk glycans with a sialic acid moiety, such as sialyllactose and glycolipid gangliosides GM1 and GM3, also have a known ability to bind pathogenic organisms, including *Escherichia coli* and *Pseudomonas aeruginosa* [28].

HMO function also includes interacting with immune cells by exhibiting similarity to selectin ligands for cell adhesion. In this manner, HMOs can bind to immune cells via selectins expressed on the cell surface and influence changes in immune cell populations and functions [29,30]. As leukocyte rolling is mediated by selectins, HMOs as selectin ligand analogs can reduce selectin ligand binding and inhibit leukocyte rolling and infiltration and subsequent inflammation and tissue injury [30].

Therefore, HMOs from breast milk have an important role in influencing the gut microbiota, shaping the integrity of the intestinal epithelium and protection of the vulnerable preterm infant.

### 2.3. HMOs and Risk of Necrotizing Enterocolitis

As human breast milk is a known protective mechanism against the development of NEC, the role of HMOs in protection against NEC has been a growing field of research. In seeking to characterize the HMO content of human breast milk, Autran and colleagues performed a multi-centered cohort analysis on the breast milk composition of 200 mothers and evaluated the risk for developing NEC in their breast fed low birth weight infants [31]. The results from this study implicate that higher breast milk concentrations of HMO disialyllacto-N-tetraose (DSLNT) is associated with a lower risk of developing NEC in the infant (Bell stage 2 and 3 combined) (OR 0.84; 95% CI 0.79 to 0.88; *p* = 0.001), although risk factors that decrease DSLNT content in mother’s milk require further investigation [31]. In preclinical studies using a newborn rat model of NEC, pups fed with a formula containing DSLNT demonstrated a reduction in NEC severity and decreased mortality [32]. The same study also showed that galacto-oligosaccharides, an additive in commercial infant formula, structurally dissimilar from natural HMOs, provided no protection against NEC or mortality in neonatal rats [32]. Similarly, a systematic review of 12 studies of prebiotics in commercial infant formula demonstrated no significant resulting change in the intestinal flora [33].

A preclinical study of neonatal mice demonstrated that supplementation with HMO-2′FL resulted in reduced experimental NEC severity with a decrease in small intestine gene expression of pro-inflammatory cytokines and protected the architecture of the small intestine [34]. Mechanistically, HMO-2′FL maintained mesenteric perfusion of the gut with preserved expression of endothelial nitric oxide synthase in the intestine [34]. A recent analysis of HMOs in breast milk from 91 mothers of extremely low birth weight infants (<1000 g) at day 14, 28, and at postmenstrual week 36 showed that the development of NEC was associated with lower levels of the fucosylated HMO lacto-N-difucohexaose (LNDH I) [35]. Additionally, low levels of HMOs sialyl-lacto-N-tetraose a (LSTa) and lacto-N-neotetraose (LNnT) at the 28-day time point were associated with the development of NEC [35]. Further analysis showed that high levels of 6′SL and low levels of 3′SL at the 28-day time point was associated with the development of sepsis [35]. In contrast, a model of NEC in preterm piglets receiving feeds with a complex blend of HMOs showed no significant difference in intestinal microbial diversity or protection against NEC [36]. Overall, these findings suggest that measurement of maternal milk for a single HMO such as DSLNT, 2′-FL, or LNDH I may provide insight into infants at higher risk for developing NEC.

## 3. Probiotics and the Preterm Infant Microbiome

Human breast milk contains an intricate community of bacterial organisms, influenced by both the maternal microbiome and maternal milk microbiome, which help shape the infant gut microbial community [37,38]. In particular, the CHILD cohort, which contained 393 breastfeeding dyads, provided insight into the composition of the maternal milk microbiome [38]. The diversity of the milk microbiome was influenced by maternal factors including the mode of breastfeeding (direct versus indirect) and mode of delivery. Indirect breastfeeding with expressed breast milk was associated with a greater abundance of *Enterobacteriaceae* and *Enterococcaceae* species and a reduced prevalence of *Bifidobacterium* species in the milk [38]. This analysis suggests that indirect breastfeeding impacts the relative abundance of potential pathogens in the milk microbiome, which may influence the development of the infant gut microbiota. Therefore, the use of enteral probiotics supplements to shape the infant microbiome remains a particular interest. Moreover, the use of probiotic supplementation for neonatal dysbiosis remains a debated topic in neonatology. Probiotics have been studied as single-strain or multi-strain live microbe supplements in both preterm and term neonates. A variety of commercial probiotic supplements are available for use, with *Bifidobacterium* and *Lactobacillus* species being the most common probiotic strains. Randomized control trials and cohort studies in the neonatal population have primarily focused on the effects of probiotic supplementation on the regulation of the host microbiome and the reduction in the risk of morbidities such as sepsis or necrotizing enterocolitis. Postulated mechanisms for the beneficial effects of probiotics were primarily developed using in vivo animal models, with few mechanistic studies existing in the neonatal population.

### 3.1. Probiotics and Protection of the Preterm Gut

Trials of probiotic supplements as a single organism and combination products in preterm infants have focused on the protective effects on the intestinal barrier and the gut microbiome. In seeking to analyze the effect of enteral probiotic strains on intestinal permeability in preterm infants, one study of 41 infants ranging from 27 to 36 weeks’ gestation, were fed with preterm commercial formula supplemented with *Bifidobacter lactis* and had reduced stool lactulose/mannitol ratios as a measure of intestinal permeability, as compared to preterm infants who received formula without *Bifidobacter* supplementation [39]. Using a mouse model to examine epithelial barrier function, Patel and colleagues identified that the enteral administration of the probiotic *Lactobacillus rhamnosus GG* induced tight junction (claudin 3) expression and reduced intestinal permeability up to 3 weeks after supplementation using serum FD4 measurements [12].

In addition to the improved structural integrity of the intestinal barrier, probiotics have also exhibited an ability to increase the diversity of the microbiome with beneficial bacteria and reduce colonization by enteric pathogens, as shown in Figure 1. In a double-blind placebo-controlled randomized study, preterm infants supplemented with *B. lactis* Bb12 had higher levels of *Bifidobacterium* in the stool just one week after treatment (*p* = 0.003) and had a reduced abundance of *Enterobacteriaceae* and *Clostridium* species [40]. Similarly, in a clinical trial of formula-fed preterm infants randomly assigned to receive two different strains of *Bifidobacterium*; the enteral supplementation with *Bifidobacterium longum subspecies infantis* (*B. longum ssp infantis*) resulted in a greater fecal Bifidobacterial colonization, although the relative *Bifidobacteria* abundance did not change with dose escalation (aOR 1.15, 95% CI 0.79–1.69). Preterm infants receiving human milk supplemented with *B. longum* ssp *infantis* demonstrated an increased fecal Bifidobacterial colonization as well as the decreased relative abundance of Gammaproteobacteria, which has been associated with pathogenicity in NEC [41].

The effects of antibiotic exposure on the gut microbiome in preterm infants in the neonatal intensive care unit (NICU) has also been well described. In particular, the administration of antibiotics in the setting of an underdeveloped immune system and a weak intestinal epithelial barrier in preterm infants has resulted in a reduced abundance of diverse gut microbial species [42,43]. The treatment of preterm infants with antibiotics has been associated with an overall decrease in total gut bacterial colonization [41]. In addition, a reduced abundance of *Bifidobacterium* and *Bacteroides* species has been shown to occur in preterm infants with a history of antibiotic exposure in the newborn period [44]. As intestinal dysbiosis in preterm infants is associated with adverse outcomes including NEC, sepsis, increased mortality, and development of antimicrobial resistance, there remains a continued need to judiciously use antibiotic therapy in the newborn period in preterm infants in the effort to prevent these significant morbidities [42,43].

### 3.2. Probiotics and Sepsis in the Preterm Infant

Intestinal dysbiosis paired with an immature immune system predisposes preterm infants to invasive bacterial infections and late-onset sepsis. Therefore, investigations of probiotic supplementation in preterm infants have placed a specific emphasis on the effects of probiotics on preventing or contributing to sepsis in this high-risk population. Several randomized control trials (RCTs) have examined a variety of probiotic combination strains to evaluate preterm infant development of sepsis. A meta-analysis of 37 RCTs analyzing probiotic use in preterm infants identified a statistically significant reduction in the risk of late-onset sepsis in infants born at less than 37 weeks’ gestation with a birth weight less than 2500 g [45]. Additionally, a network meta-analysis by van den Akker and colleagues, which included 51 independent studies, concluded that two specific complex strain combinations provided a reduced relative risk of late-onset bacterial sepsis in a cohort of preterm infants: *B. bifidum, B. infantis, B. longum,* and *L. acidophilus* as well as the combination of *B. longum R00175*, *L. helveticus R0052*, *L. rhamnosus R0011*, and *S. boulardii CNCM I-1079* [46].

Invasive fungal infections (IFIs) also provide a significant threat to the survival of preterm infants. IFIs are associated with significant morbidity and neurodevelopmental impairment, with a mortality rate ranging 30–75% [47]. Manzoni and colleagues demonstrated the benefits of enteral supplementation with *L. casei* subspecies *rhamnosus* on the prevention of enteric colonization and development of sepsis by *Candida* species in preterm and very low birth weight infants (<1500 g) in the NICU [47].

These analyses, however, contrast the outcomes of several large multi-center trials [48,49,50]. The ProPrems trial in 2013 with 1099 subjects used a combination probiotic containing *B. infantis*, *Streptococcus thermophilus*, and *B. lactis* administered to preterm infants with a birth weight less than 1500 g [49]. This group found no significant difference in the risk of late-onset sepsis in the group less than 32 weeks’ gestation [49], which has been attributed to the small sample size of the study [45]. However, the study group did identify a decreased incidence of late-onset sepsis in infants ≥28 weeks’ gestation and additionally identified a reduced risk of developing Bell stage 2 NEC or greater (RR 0.46, 95% CI 0.23 to 0.93) [49]. The PiPS Trial, which enrolled infants less than 31 weeks’ gestation, also identified no significant decrease in the risk of late-onset sepsis or NEC in infants receiving single-strain probiotic supplementation [51].

In summary, combination strain probiotics may have a beneficial effect in decreasing the incidence of late-onset bacterial and fungal sepsis in preterm infants. However, a multi-center RCT with a large sample size remains necessary in order to provide sufficient evidence, safety, and quality of a probiotic supplement to identify the most efficacious approach to using probiotics as an effective therapeutic strategy for neonatal sepsis [48].

### 3.3. Probiotics and the Prevention of Necrotizing Enterocolitis

The composition of the neonatal gut microbiome and dysbiosis remains an important risk factor for the development of NEC. The dysbiosis that precedes NEC is characterized by a bloom of intestinal Gammaproteobacteria and can be exacerbated after exposure to antibiotics [5,10,52]. Hence, most probiotic trials in infants have provided a focus on the changes in gut microbial colonization by probiotics to assess for the prevalence of pathogenic bacteria that may contribute to the development of NEC.

The use of probiotics for the prevention of NEC was first described two decades ago in the Hospital Simon Bolivar, in Bogotá, Colombia. In this study, *Lactobacillus acidophilus* and *B. infantis* at a dose of 2.5 × 10^8^ of each organism were given to 1237 infants matched with historical controls and resulted in a reduced incidence of NEC (*p* < 0.0005) and a decrease in NEC-related mortality (*p* < 0.0005) [53]. The relative risk for NEC in preterm infants has since been shown to be significantly reduced in probiotic formulations containing a combination of *Lactobacillus* and *Bifidobacterium* strains, although the optimal strains, timing, and dosing have yet to be identified [46,54,55].

In determining specific beneficial strains of *Bifidobacteria*, *B. bifidum* supplementation has been shown in one analysis to be protective against inflammation and to reduce the risk of NEC, whereas others have shown *B. infantis* provides attenuation of inflammation in a rat model of NEC [56,57,58,59]. A 2014 Cochrane Review concluded that probiotics are effective in preventing severe NEC and all-cause mortality in preterm and very low birth weight infants but could not identify a specific strain with the best effect. Specifically, probiotic preparations containing *Lactobacillus* alone, or in combination with *Bifidobacterium* strains, were shown to reduce the incidence and severity of severe NEC (Figure 1) with no reduction in sepsis [60]. However, some probiotic strains, such as *L. reuteri ATCC 55730* may play a role in improved gastric motility and feeding tolerance, thereby preventing the intestinal dysbiosis that precedes NEC [11]. Olsen and colleagues provided a systematic review and meta-analysis of 12 studies comprising 10,800 preterm or low birth weight infants receiving prophylaxis with primarily combination strains containing *Lactobacillus* and *Bifidobacterium* who experienced a significantly decreased risk of NEC (RR = 0.55, 95% CI, 0.39–0.78; *p* = 0.0006) and overall mortality (RR = 0.72, 95% CI, 0.61–0.85; *p* < 0.0001) when compared to healthy controls [61], supporting the growing evidence for probiotics as a preventative strategy in NEC. A recent large network meta-analysis of 63 RCTs and 15,712 preterm, low birth weight infants compared the effectiveness of single versus multiple strain probiotics [62]. This meta-analysis concluded that a combination of one or greater strains of *Lactobacillus* species and one or greater strains of *Bifidobacterium* species are the most efficacious in the prevention of all-cause mortality and NEC Bell stage 2 [62]. Additional evidence suggested that *Bacillus* species, *Enterococcus* species, *Bifidobacterium* species, and S. *thermophilus* are also effective in preventing stage 2 NEC [62].

Although comprehensive meta-analyses of RCTs and observational studies have demonstrated the beneficial role of probiotics in the prevention of NEC and mortality [61], several studies, including large multi-center RCTs, have not demonstrated this preventative effect [48,63]. Due to the overall heterogeneity in the probiotic strains used in the studies, the use of single versus combination strains, and the variation in feeding strategy, further investigation is required for the identification of the most effective approach to the use of probiotics in this population.

### 3.4. Risks of Probiotics

One particular area of focus is the safety of probiotic use in preterm infants. Currently, the United States Food and Drug Administration (FDA) and the American Academy of Pediatrics have not promoted the administration of probiotics to preterm infants [64]. There remains a concern regarding the theoretical risk of bacterial translocation of probiotic organisms due to immaturity of the immune system and immaturity of the intestinal barrier function in preterm infants [64]. Therefore, there is continued hesitancy in recommending routine use of probiotic supplementation in preterm infants until regulated products and large efficacy trials become readily available. Additionally, as probiotics in the United States are typically regarded as food supplements rather than pharmaceutical products, the limited regulation of probiotics place them at risk for contamination with harmful pathogens [64]. This has been previously described by the Centers for Disease Control in 2015 after a preterm infant developed a fatal case of gastrointestinal mucormycosis after receiving an enteral combination-strain probiotic. In this case, the probiotic supplement yielded growth of *Rhizopus* species on microbial culture, and the same species was identified upon DNA sequencing of a resected intestinal tissue specimen from the infant [65]. Adverse effects of probiotics, namely probiotic-associated bacteremia or sepsis, have been reported in case reports [66,67,68,69] as well as in a recent analysis of *Lactobacillus* bacteremia in intensive care units (ICUs) [70]. In this analysis, 522 critically ill neonatal and pediatric patients treated with *L. rhamnosus* strain GG probiotics demonstrated a higher risk of developing *Lactobacillus* bacteremia than patients receiving no probiotic supplementation [70]. Genome-level analysis of the six ICU patients with *Lactobacillus* bacteremia confirmed that the isolated strains from patient blood samples and the *Lactobacillus* probiotic supplement were identical [70]. These patients did not exhibit the characteristic risk factors, such as gastrointestinal dysfunction or an immunocompromised state, that predispose an individual to the development of probiotic-related complications [70]. Overall, despite the potential benefits of probiotics, this study provides caution in treating ICU patients with probiotics due to the risk of probiotic-associated bacteremia.

Taken together, the current evidence supports the benefits of probiotics in strengthening the immature intestinal barrier to reduce the incidence and possibly the severity of NEC in a particular population of preterm infants. Additional knowledge should be obtained to translate this evidence into the recommended use of specific probiotic strains in preterm infants, provided that large scale and multi-centered validation studies confirm their safety and efficacy.

## 4. Synbiotics

As HMOs and probiotics have been separately identified to have a benefit in the infant gut microbiome, reduced risk of morbidities such as NEC, and long-term impact on neurodevelopment, recent studies have examined the synergistic effects of these agents when supplemented in combination. Studies on the concurrent use of prebiotic components and probiotic microbes is limited in the neonatal population and in the setting of NEC. Although maternal antenatal supplementation with synbiotics (inulin in combination with *L. sporogenes*) has been trialed and demonstrated improved serum insulin levels, the subsequent effects on newborns were not investigated [71].

Dilli and colleagues performed an RCT of 100 infants ≥35 weeks’ gestation with cyanotic congenital heart disease using synbiotic supplementation containing *Bifidobacterium* plus inulin and showed a significant reduction in NEC incidence, development of sepsis, and mortality [72]. In a separate prospective multi-centered RCT in 677 very low birth weight infants, Dilli and colleagues used this same synbiotic supplement and demonstrated a reduced rate of NEC Bell stage 2 or greater (4.0%) and reduced rates of mortality (*p <* 0.003) when compared with placebo (18.0%) (*p* < 0.001) [73].

Underwood and colleagues showed that single and combination probiotic strains in synbiotic preparations (*Lactobacillus* plus fructooligosaccharides (FOS), *Lactobacillus* and *Bifidobacterium* plus FOS) increased the stool abundance of *Bifidobacterium* by 100,000-fold but had no impact on the incidence of NEC or growth outcomes when compared with a placebo [74]. The synbiotics also did not affect the short-chain fatty acid content, the presence of which in stool serves as a measure of colonic pH and risk of intestinal mucosal injury [74]. Comparatively, the use of a similar synbiotic containing *Lactobacillus* and *Bifidobacterium* plus FOS was trialed in 220 preterm infants (delivered at 28–34 weeks’ gestation and >1000 g birthweight) and showed no difference in outcomes related to the incidence of NEC, sepsis, or mortality when compared with controls [75]. However, in stool cultures, increased gut colonization by *Lactobacilli* was noted after 7 days of synbiotic supplementation [75]. The use of a complex synbiotic preparation containing the combination-strain probiotics *L. acidophilus*, *B. longum*, *B. bifidum*, *S. thermophiles,* plus FOS was administered to infants ≤34 weeks’ gestation receiving enteral feeds with breast milk only [76]. In this analysis, the development of NEC and sepsis was significantly decreased when compared to breast fed controls without synbiotic supplementation [76]. A prospective, double-blinded RCT was performed in very low birth weight infants ≤32 weeks’ gestation and <1500 g birth weight receiving enteral feeds with a synbiotic combination (*L. rhamnosus*, *L. plantarum*, *L. casei*, *B. lactis* plus FOS, and galactooligosaccharide) in addition to lactoferrin and vitamins versus placebo [77]. This analysis yielded a reduced incidence in all stages of NEC with no difference in the incidence of late-onset culture-positive sepsis [77].

Overall, RCTs of preterm infants receiving single-strain or multi-strain synbiotics exhibit great variability in the probiotic strain and dosage as well as variability in the prebiotic oligosaccharide of choice. Additionally, mechanisms for the proposed benefits of synbiotics remains undefined. As the outcomes in these studies remain conflicting, the use of synbiotics in preterm infants requires ongoing investigation for both safety and efficacy.

## 5. Conclusions

Preterm infants are vulnerable to intestinal dysbiosis and an immature intestinal barrier that predisposes them to morbidities such as sepsis and NEC. Human breast milk contains an assortment of bioactive components that are required by preterm infants for adequate growth as well as intestinal and immune maturation. HMOs are present in abundance in breast milk and have been shown to influence the gut microbiota and the integrity of the intestinal epithelial barrier, implicating a potential ability to provide protection against inflammation and the development of NEC. Enteral probiotic supplements have also been shown to have a protective role in influencing the composition of the gut microbiota with beneficial and commensal organisms. A variety of RCTs have used single and combination strain probiotics to demonstrate the effects on preterm infant gut barrier dysfunction and prevention of developing sepsis and NEC, though large scale and multi-centered validation studies are required to confirm the safety and validity of probiotic supplementation in this vulnerable population. The growing field of research studying prebiotic HMOs and probiotics reinforces the immunological significance of human milk and its impact on infant nutrition and outcomes.
